# Enhanced accumulation of N-terminally truncated Aβ with and without pyroglutamate-11 modification in parvalbumin-expressing GABAergic neurons in idiopathic and dup15q11.2-q13 autism

**DOI:** 10.1186/s40478-020-00923-8

**Published:** 2020-04-28

**Authors:** Janusz Frackowiak, Bozena Mazur-Kolecka, Pankaj Mehta, Jerzy Wegiel

**Affiliations:** Department of Developmental Neurobiology, NYS Institute for Basic Research in Developmental Disabilities, Staten Island, New York, NY10314 USA

**Keywords:** Idiopathic autism, Dup15q11.2-q13 autism, Prefrontal cortex, GABAergic interneurons, Parvalbumin-expressing interneurons, Somatostatin-expressing interneurons, N-terminally truncated amyloid-β peptide, Pyroglutamate-11 Aβ modification

## Abstract

Autism, the most frequent neurodevelopmental disorder of a very complex etiopathology, is associated with dysregulation of cellular homeostatic mechanisms, including processing of amyloid-β precursor protein (APP). Products of APP processing — N-terminally truncated amyloid-β peptide (N-tr-Aβ) species — are accumulated in autism in neurons and glia in the cortex, cerebellum, and subcortical structures of the brain. This process in neurons is correlated with increased oxidative stress. Because abnormally high levels of N-tr-Aβ are detected in only a fraction of neurons in the prefrontal cortex, we applied immunocytochemical staining and confocal microscopy in autopsy brain material from idiopathic and chromosome 15q11.2-q13 duplication (dup-15) autism to measure the load of N-tr-Aβ in the cells and synapses and to identify the subpopulation of neurons affected by these pathophysiological processes. The peptides accumulated in autism are N-terminally truncated; therefore, we produced a new antibody against Aβ truncated at N-terminal amino acid 11 modified to pyroglutamate to evaluate the presence and distribution of this peptide species in autism. We also quantified and characterized the oligomerization patterns of the Aβ-immunoreactive peptides in autism and control frozen brain samples. We provide morphological evidence, that in idiopathic and dup-15 autism, accumulation of N-tr-Aβ with and without pyroglutamate-11 modified N-terminus affects mainly the parvalbumin-expressing subpopulation of GABAergic neurons. N-tr-Aβ peptides are accumulated in neurons’ cytoplasm and nucleus as well as in GABAergic synapses. Aβ peptides with both C-terminus 40 and 42 were detected by immunoblotting in frozen cortex samples, in the form of dimers and complexes of the molecular sizes of 18-24kD and 32-34kD. We propose that deposition of N-tr-Aβ specifically affects the functions of the parvalbumin-expressing GABAergic neurons and results in a dysregulation of brain excitatory–inhibitory homeostasis in autism. This process may be the target of new therapies.

## Introduction

Autism is a neurodevelopmental disorder characterized by impaired interpersonal communication and stereotyped and repetitive behaviors. The etiology and pathogenesis of autism are not known, but a combination of genetic, epigenetic, and environmental factors, has been implicated in autism. Autism is associated with dysregulation of certain basic cellular homeostatic mechanisms, as indicated by altered processing of amyloid-β precursor protein (APP) detected in children with autism. Plasma levels of secreted APP-α, but not APP-β, were reported to be two or more times higher in children with severe autism and aggressive behavior than in children without autism, and up to four times higher than in children with mild autism [38, 43]. In another study, the levels of secreted APP-α in plasma were found to be significantly increased in 60% of autistic children, as compared to age-matched controls [2]. It has been proposed that increased processing of APP by secretases-α may contribute to development of autism symptoms [38].

Neurons and glia in the brain cortex, cerebellum, and subcortical nuclei in autism accumulate amino-terminally truncated amyloid-β peptide (N-tr-Aβ) — corresponding to a product of secretase-α and secretase-γ [48]. The abundance of these deposits in neurons correlates with accumulation of lipid oxidation derivatives: MDA and 4-HNE [16]. Based on these findings, we hypothesized that intraneuronal accumulation of N-tr-Aβ in autistic children increases the formation of oxygen free radicals that cause lipid peroxidation and lead to a further formation of Aβ in a self-enhancing vicious circle contributing to neuron dysfunction in autism.

Numerous clinical symptoms in autism, as well as the increased prevalence of epilepsy, suggest a dysfunction of the inhibitory GABAergic system. Several studies indicate that genetic predisposition to autism may be associated with the genes coding for the GABA(A) receptor. The chromosome 15q11-q13 region containing three GABA receptor subunit genes is an autism candidate region [41]. Production of the GABA neuromediator is catalyzed by two isoforms of glutamic acid decarboxylases (GAD): GAD67, present in perikaryon and regulated by neural activity, and GAD65, present in synapses [29]. In autism downregulation of both GAD67 and GAD65 to 50% of the levels of controls, has been reported in the parietal and cerebellar cortices, dentate nucleus, and amygdala [7, 13].

The prefrontal cortex contains two neuronal populations: glutamatergic excitatory neurons and GABAergic inhibitory interneurons, both of which contain cells of heterogenous morphology and functions. The most numerous subpopulations of the GABAergic interneurons in neocortex are the cells that express the Ca2 + −binding protein parvalbumin (PVA), the neuropeptide somatostatin (SST), and the ionotropic serotonin receptor 5HT3a. These populations have different embryological origins and distinct functional properties. The most frequent PVA expressing interneurons which include basket cells, and chandelier cells, represent about 40 to 50% of GABAergic neurons, and the cells expressing SST and 5HT3a receptors represent about 30% and 20–30% of GABAergic neurons, respectively. These GABAergic subpopulations represent heterogeneous groups that contain cells that can be differentiated by expression of the neuropeptide VIP and other neuropeptides. Distinct neuronal subgroups are probably involved in modulating cortical circuits during specific behavioral tasks and contexts (reviewed in: [22, 23, 40]). Abnormalities and dysfunctions of specific interneuron populations may result in distinct developmental neurological disorders.

Previous studies demonstrated that enhanced accumulation of Aβ-immunoreactive material was limited to a fraction of neurons in distinct brain structures [48] and that in the prefrontal cortex, only a fraction of neurons accumulated Aβ-immunoreactive material and lipid peroxidation products [16]. Thus, the aim of this study was to identify the subpopulations of neurons affected by these pathophysiological processes in idiopathic autism and in chromosome 15 duplication with autism (dup-15). Because Aβ accumulated in the brain in autism appears to be N-terminally truncated [16, 48], and the main product of α-secretase cleavage of APP is the peptide with N-terminal aminoacid-11 — glutamic acid — we also tested the hypothesis that neurons in autism accumulate pyroglutamate-11–modified Aβ. In this study, we provide morphological evidence that abnormal accumulation of N-tr-Aβ with and without pyroglutamate-11 modified N-terminus in autism affects mainly the subpopulation of GABAergic neurons expressing parvalbumin, but not those expressing somatostatin.

## Materials and methods

### Tissues

Postmortem formalin-fixed prefrontal cortex samples from individuals with idiopathic autism (*n* = 6), dup-15 autism (*n* = 7) and controls (n = 6), as shown in Table 1, were obtained from the Brain and Tissue Bank for the Developmental Disabilities and Aging at IBRDD, Staten Island, The Harvard Brain Tissue Resource Center (R24-MH068855) and the Brain and Tissue Bank at the University of Maryland, Baltimore, MD. Diagnosis of autism was confirmed by the Autism Diagnostic Interview – Revised (ADI-R). The diagnosis of dup-15 for all the cases was confirmed by genotyping with 19–33 short tandem repeat polymorphisms from chromosome 15, Southern blot analysis of dosage with 5–12 probes and by fluorescent in situ hybridization performed using antemortem peripheral blood samples and lymphoblast cell lines. The tested individuals were tetraploid for the chromosome 15 segment except for one person who was hexaploid [32, 46]. The brain tissues were embedded in polyethylene glycol and cut into 50 μm thick sections for immunofluorescence and confocal microscopy studies or were frozen and kept at -70 °C for biochemical analysis.

**Table 1:** Formalin-fixed brains examined

Group	Brain Bank number	Sex	Age years	Cause of death	PMI
Dup-15	AN14762	M	9	SUDEP	13.6
Dup-15	AN06365	M	10	SUDEP	17.7
Dup-15	AN09402	M	11	SUDEP	10.5
Dup-15	AN07740	F	15	SUDEP (suspected)	24
Dup-15	AN09470	F	15	Pneumonia	–
Dup-15	AN03935	M	20	Cardiac arrest, choking	28
Dup-15	AN05983	M	24	SUDEP	36
Autism	HSB4640	M	8	Asthma attack	13.8
Autism	AN01293	M	9	Cardiopulmonary arrest	3.8
Autism	CAL105	M	11	Drowning	–
Autism	AN11206	M	15	Asphyxia	38
Autism	IBR93–01	M	23	Seizure related	14
Autism	NP06–54	M	32	Glioblastoma	–
Control	UMB1706	F	8	Rejection of cardiac transplant	20
Control	CNL1548	M	10	Carbon monoxide poisoning	–
Control	UMB1670	M	14	Asphyxia (hanging)	5
Control	UMB4722	M	14	Multiple traumatic injuries	20
Control	BTB-3960	F	25	Not known	26
Control	CNL1169	M	32	Heart failure	14

NOTE: postmortem interval (PMI); Sudden unexpected and unexplained death of subject with known epilepsy (SUDEP)

### Generation, purification and testing antibody against pyroglutamate-11 Aβ

Previous studies showed abnormal accumulation of N-terminally truncated Aβ in the brain in autism [16, 48]. Aβ peptide with N-terminal glutamic acid-11 may become modified to pyroglutamate, hence, we produced a new antibody against Aβ truncated at N-terminal aminoacid 11 modified to pyroglutamate.

The peptide Glp-VHHQKL-C6-C (American Peptide Company, Sunnyvale, CA, USA) conjugated to keyhole limpet hemocyanin was used to immunize rabbits, as approved by the Animal Welfare Committee IACUC, the sera were affinity purified and the specificity of the antisera was tested by indirect ELISA and dot blotting, as previously described [33]. The peptides used as standards were: synthetic Aβ-pE11–40 (Bachem Americas, Inc., Torrance, CA, USA), Aβ-pE11–42 (AnaSpec Inc., Fremont, CA, USA, and Rockland Inc. Limerick CA, USA), Aβ-11–40 (rPeptide, Bogart, GA, USA), Aβ-11–42 (American Peptide Co., Sunnyvale, CA, USA), Aβ-1-40, and Aβ-1-42 (Bachem). The stock solutions of peptides in hexafluoro-2- propanol (Sigma, St. Louis, MO), 1 mg/ml were dispersed in an ultrasonic disintegrator, diluted in water. For dot blotting the peptides were applied onto a 0.1 μm nitrocellulose membrane (Whatman GmbH, Dassel, Germany), as described previously [15, 33]. The peptide Aβ-pE11–42 was oligomerized after dilution the stock solution in PBS [19]. The untreated or boiling-denatured membranes were probed with affinity purified antibodies against Aβ-pE11 at the concentration of 0.05 and 0.2 μg/ml. As reference antibodies mouse mAb 6E10 and mAb 4G8 were used. The reactions were developed using goat anti-rabbit IgG conjugated to alkaline phosphatase as described previously [15, 33].

### Immunofluorescence and confocal microscopy

Aβ and neuronal markers were detected with the panel of antibodies listed in (Table 2), in sections of prefrontal cortex containing Brodmann cortical areas 9, 44, 45 and 46 by the indirect immunofluorescence and confocal microscopy, as previously described [16, 48]. GABAergic neurons and synapses were identified by immunostaining for GAD67 and GAD65. Because layers 2–6 of prefrontal cortex contain two major classes of neurons: glutamatergic pyramidal neurons making 70–80% of the total neural population and up to 30% of GABAergic interneurons [9], the neurons not reactive for GAD65/67 could be identified as glutamatergic by their morphology in sections counterstained with TO-PRO-3-iodide (TOPRO-3i) (Invitrogen/Molecular Probes).

**Table 2:** Antibodies used for immunohistochemistry and for immunoblotting

Name	Epitope or target	Dilution	Host/type	Source
6E10	4–10 aa Aβ	1:4000	M-monocl	IBRDD [19, 26]
4G8	18–23 aa Aβ	1:3000	M-monocl	IBRDD [19, 26]
MOAB-2	Aβ ^a^, not APP	1:100	M-monocl	LifeSpan Biosciences, Inc., Seattle, WA
IBR226	Aβ C-terminus 36–42	1:40	R-polycl	IBRDD [14]
12F4	Aβ-42 C-terminus	1:200	M-monocl	BioLegend, San Diego CA
RabmAb42	Aβ C-terminus 36–42	1:100	R-monocl	IBRDD [34]
IBR162	Aβ C-terminus 34–40	1:200	R-polycl	IBRDD [14]
R510	Aβ-pE11	1:2000	R-polycl	IBRDD
R57	APP aa 671–695	1:2000	R-polycl	IBRDD [14, 15]
Anti-GAD65/67	glutamic acid decarboxylase	1:250	R-polycl	Millipore-Sigma, Burlington, MA
Anti-parvalbumin	parvalbumin	1:100	S-polycl	ThermoFisher, Waltham, MA
Anti-parvalbumin	parvalbumin	1:20	G-polycl	Novus Biologicals, LLC, Centennial, CO
Anti-somatostatin	somatostatin	1:100	S-polycl	Novus Biologicals, LLC, Centennial, CO
Ab-1	cathepsin D	1:200	M-monocl	EMD Biosciences, San Diego, CA

^a^ Unaggregated, oligomer and fibrillar Aβ, no cross-reaction with human APP

The antibodies were monoclonal or polyclonal affinity purified mouse (M); rabbit (R) sheep (S) or goat (G)

To identify Aβ accumulation in GABAergic neurons’ subpopulations sections were triple immunostained with a rabbit antibody against GAD65/67, goat or sheep antibodies against PVA or SST (Table 2), and mouse antibody against Aβ (mAb 4G8). Secondary antibodies were affinity-purified donkey antibodies against mouse and rabbit IgG labelled with Alexa488 and Alexa555 (Invitrogen/Molecular Probes, Grand Island, NY, USA) and donkey anti-goat/sheep labelled with Alexa647 (Invitrogen/Molecular Probes) or with NL637 (R&D Systems). Nuclei were not counterstained but the cell contours and boundaries were discernible in the phase contrast channel and as nonspecific fluorescence in the green, red and blue channels. Glutamatergic neurons were identifiable among GABAergic synapses and their contours could be delineated in the images collected with enhanced background in all channels versus blood vessels and other cells.

Images were collected using a Nikon C1 confocal microscope system and with EZC1 image analysis software. The images were used for further immunofluorescence quantification with Image J software (NIH), based on previous studies that have shown that measurements of immunofluorescence staining allow relative protein quantification in tissue sections when properly standardized methods are used [1, 16]. The guidelines for proper image acquisition and controlling factors that affect the accuracy and precision of quantitative fluorescence microscopy were applied [47]. Sections were coded and microscopic fields of 0.101mm^2^ in the 3rd, 4th and 5th cortical layers were randomly selected in the blue channel in which only cell nuclei and cytoplasm were visible, for unbiased sampling. Magnified areas used for measurements were between 6600μm^2^ and 16200μm^2^. The images were collected in three channels with the channel amplification settings the same for all groups tested. Specificities of immunostainings were confirmed as previously described [15, 16, 48]. Specific immuofluorescence was obtained by subtracting autofluorescence and nonspecific background fluorescence, as previously reported [16]. The levels of specific immunofluorescence intensities for Aβ, Aβ-pE11, GAD65, GAD67, parvalbumin and somatostatin were calculated and expressed in arbitrary units (AU) for the cell and nucleus contours and in random 100μm^2^ samples of surrounding neuropil without blood vessels. For each tested individual and each immunostaining an average of 38 cells were measured, and 50 to 60 cells for triple immunostainings.

### Characterization of Aβ accumulation by immmunoblotting

Frozen samples of prefrontal cortex from 3 control, 1 idiopathic autism and 1 dup-15 subjects (Table 3) were homogenized in a glass-teflon homogenizer in RIPA buffer with protease inhibitor cocktail (Roche Diagn. GmbH, Mannheim, Germany). Blood vessels and leptomeninges were removed by passing through 75 μm nylon mesh, and protein content was assayed by the BCA method (Thermo Scientific Pierce, Waltham, MA, USA). Samples of lysates were subjected to sequential centrifugation to separate cellular structures of different sizes, at 1000 g for 5 min, the supernatants further centrifuged at 16,000 g for 10 min, and finally the supernatants centrifuged at 100,000 g, for 30 min, and the pellets 1, 2 and 3, and supernatant 3 were collected.

**Table 3:** Frozen brains examined

Group	Brain Bank number	Sex	Age years	Cause of death	PMI
Dupl-15	AN03935	M	20	Cardiac arrest, choking	28
Autism	HSB4640	M	8	Asthma	13.8
Control	B-5251	M	19	Pneumonia	18.6
Control	UMB-818	M	27	Accident - multiple injuries	10
Control	CNL-1169	M	32	Congestive heart failure	14

NOTE: postmortem interval (PMI)

Full lysates containing 40 μg of proteins, as well as pellets 1, 2 and 3 and supernatant 3 obtained from 40-μg protein samples of full lysate, were subjected to PAGE on 8–15% gradient gels, electro-transferred onto 0.1 μm pore nitrocellulose (Whatman GmbH, Dassel, Germany) and probed with antibodies IBR162 and IBR226, specific for Aβ40 and Aβ42 C-terminal sequences, respectively, and which do not react with APP or larger APP fragments [14, 15]. The reactions were developed with secondary AP-conjugated antibodies and BCIP and NBT, and semi-quantified by densitometry. Densitometrical measurements of bands on membranes were performed with Image J software (NIH).

### Statistical analysis

The data groups were analyzed for the degrees of asymmetry of the data distribution around mean values. Because the data did not have a normal distribution (measured as skewness of the data distribution) natural logarithms of values were used for the Student’s t-test analysis. Comparisons were calculated using Student’s t-test adjusted for the non-homogeneity of variance between two groups. Correlations between the measured cell parameters were evaluated by calculating Pearson’s correlation coefficient.

## Results

### Intraneuronal amino-terminally truncated Aβ in immunohistochemical detection

**Fig. 1:**
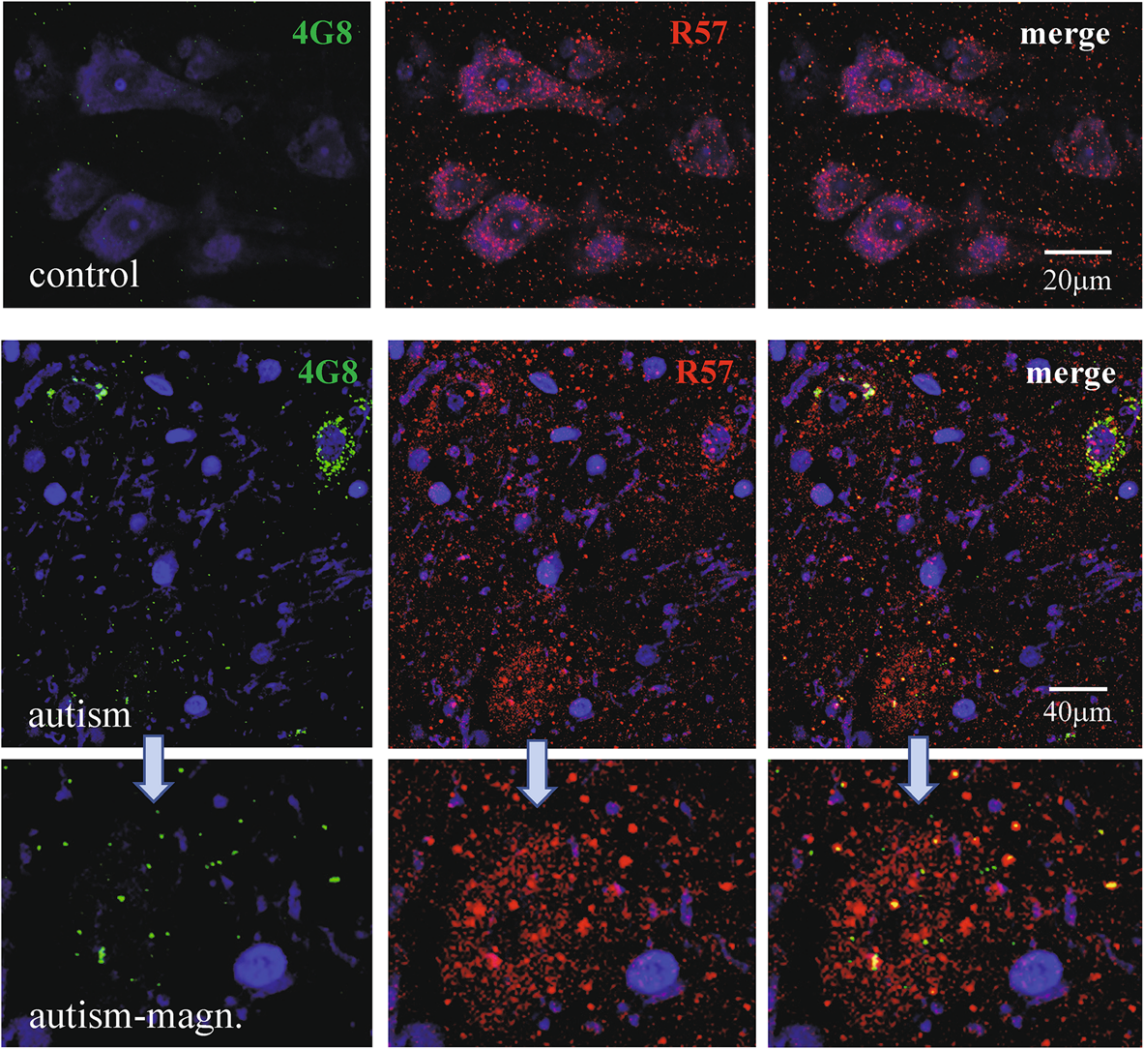
Prefrontal cortex in 8-year-old control and 11-year-old autistic subjects immunostained with C-terminal APP-specific antibody R57 (red color), mAb 4G8 (green color) and nuclei counterstained blue with TOPRO-3i. The neurons in the control contain numerous R57 immunoreactive granules and very scanty reaction with mAb4G8. In autism R57 immunoreactive material in distinct neurons is either partially 4G8-positive or mostly 4G8-negative, while only a fraction of 4G8-positive granules is positive for C-terminal APP, as shown in the magnified image (autism-magn.)

Sections from control brains immunostained with the antibody R57 specific for C-terminal APP contained multiple intracellular granules while there were only minimal reactions with mAb 4G8 (Fig. 1) and 6E10 (not shown) and no reaction with MOAB2 (Aβ-specific, not shown). In autistic subjects, the immunoreactivity with mAb 4G8 showed variable intensity in individual neurons, ranging from negligible to strong. Most of the APP immunoreactive material shown by R57 antibody was 4G8-negative and most of the 4G8-positive granules were negative for APP (Fig. 1). In the sections from autism that showed an abundant reaction with mAb 4G8 and with mAb MOAB-2 specific for Aβ (Fig. 9) there was no immunoreaction with mAbs 6E10 (not shown). The immunoreactions with mAbs 12F4 and RabmAb42 specific for Aβ42 C-terminus (not shown) indicated that at least a fraction of the peptides in the deposits contained the Aβ42 C-terminus. The 4G8-immunoreactivity was present in neurons with scanty and abundant lipofuscin autofluorescent granules (Suppl. Fig. 1) and individual cortical neurons contained: (1) Aβ immunoreaction in autofluorescent granules, (2) Aβ immunoreaction not related to lipofuscin and (3) autofluorescent lipofuscin granules without reaction for Aβ. The intraneuronal autofluorescent granules of lipofuscin were not immunoreactive with mAb 6E10 (Suppl. Fig. 1) while the antibodies specific for the C-terminus of Aβ42 — mAb 12F4 and polyclonal (pAb) R226 — immunoreacted with less than 60% of autofluorescent granules (not shown).

These results show that the immunoreactivity detected with mAb 4G8 in the autism brain sections is consistent with the distribution and amount of N-tr-Aβ but not APP and is in part located in lipofuscin. Autofluorescence of lipofuscin as well as nonspecific background fluorescence in further studies were subtracted from measurements of the specific immunofluorescence.

### Characterization of the Aβ-immunoreactive material by immunoblotting

**Fig. 2:**
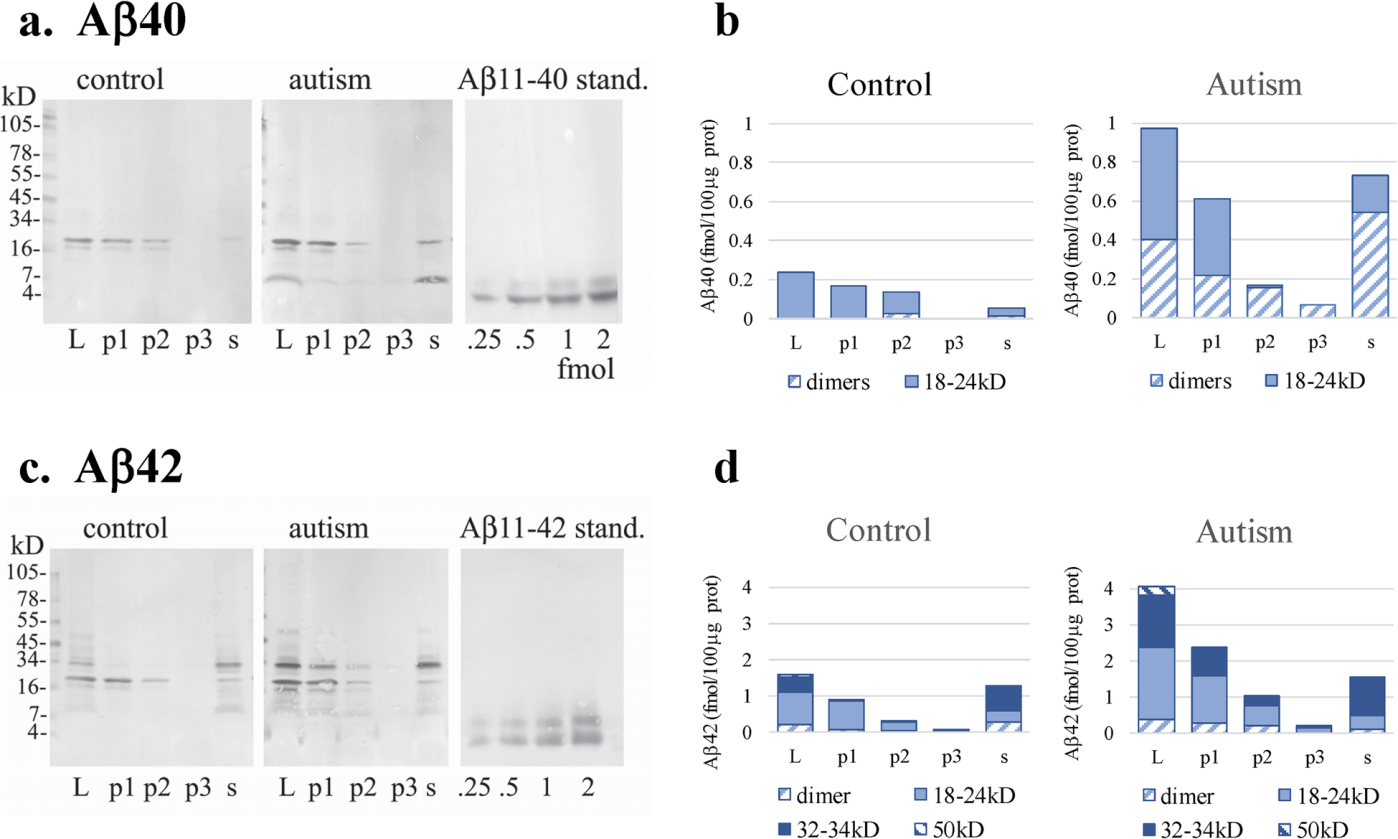
Prefrontal cortex lysates, pellets and supernatant from sequential centrifugations performed in samples from control and autism cortex samples. The contents of the Aβ40– (**a**, **b**) and Aβ42–immunoreactive peptides (**c**, **d**) were significantly higher in the autism than in the control cortex. The Aβ40 peptides in autism were detected as dimers and 18-24kD protein bands, the latter present mainly in pellet 1, containing larger subcellular structures. Aβ42 was detected mainly as 18-24kD and 32-34kD with a minor contribution of dimers and a 50-52kD protein band, all of which were more abundant in autism. Pellets 1 and 2 contained most of the Aβ42 complexes of molecular sizes 18-24kD and 32-34kD. The majority of the Aβ42 complexes of molecular sizes 32-34kD were detected in the soluble fraction (supernatant 3). The graphs (**b, d**) show average values from 3 experiments

In order to evaluate the contribution of Aβ peptides with C-terminal aa40 and aa42 to deposits of various sizes, frozen prefrontal cortex control and autism samples were lysed and subjected to sequential centrifugation. The procedure yielded pellet 1 (1000 g, 5 min) that contained 36% of the total lysate proteins, pellet 2 (16,000 g, 10 min) — 16%, pellet 3 (100,000 g, 30 min) — 4%, and supernatant — 44% of proteins, respectively. Prefrontal cortex samples contained the Aβ40 and Aβ42 species as dimers and several distinct complexes/oligomers which were SDS stable, of molecular sizes between 18kD and 52kD, both in control and in autism. The reactions for both Aβ40 and Aβ42 were more intense in autism, and the calculated total Aβ content per 100 μg of total proteins was 2.4–2.8 times higher in autism than in control (Fig. 2).

The Aβ40-immunoreactive peptides in control were present in the form of SDS-stable complexes of the molecular size 18-24kD which were recovered in pellets 1 and 2, as well as small quantities of dimers (Fig. 2a). In autism most of Aβ40-immunoreactive peptides were detected in the 18-24kD complexes recovered in pellets 1 and 2, and there was also a significant fraction of dimers that were soluble and recovered in the 100.000 g supernatant (Fig. 2a, b). The Aβ42-immunoreactive peptides were detected mainly in SDS-stable complexes of the molecular sizes 18-24kD and 32-34kD which were more abundant in autism, particularly the latter (Fig. 2c, d). Small quantities of dimers and 50-52kD complexes were also detected. The 18–24 kD complexes were present mainly in larger subcellular structures (pellet 1), and less in pellets 2, while the 32–34 kD complexes and Aβ42 dimers were mainly found in the soluble fraction. The sequential centrifugation revealed that larger and medium sized subcellular structures contained mainly Aβ42 complexes of molecular sizes 18-24kD and 32-34kD and peptides with the C-terminus 40 as complexes of molecular size 18-24kD.

### N-tr-Aβ in GABAergic and glutamatergic neurons

**Fig. 3:**
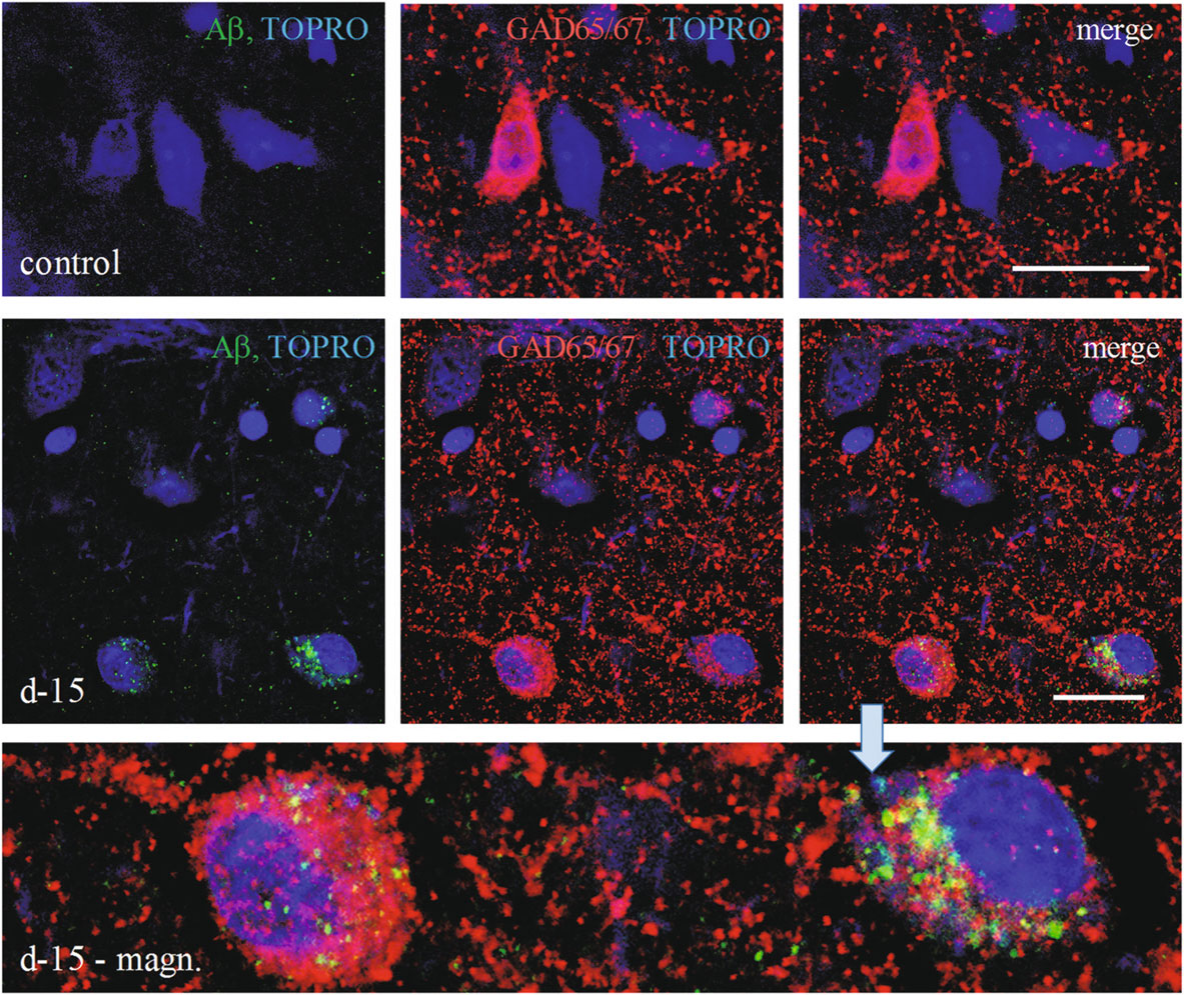
Control and dup-15 (d-15) sections double immunostained for GAD65/67 and for N-tr-Aβ (Aβ) with mAb 4G8 and counterstained with TOPRO-3i (TOPRO). The immunoreaction for N-tr-Aβ was more intense and with more numerous intracellular profiles in dup-15 subjects than in controls. The reaction for Aβ was mainly detectable in the GAD65/67-positive neurons in all studied groups as cytoplasmic granules, with a notable granular reaction in the nucleus, as shown in the magnified inset (d-15 – magn.). GABAergic neurons with a strong N-tr-Aβ immunoreaction typically showed a less intense reaction for GAD65/67. The scale bars show 20 μm

**Fig. 4:**
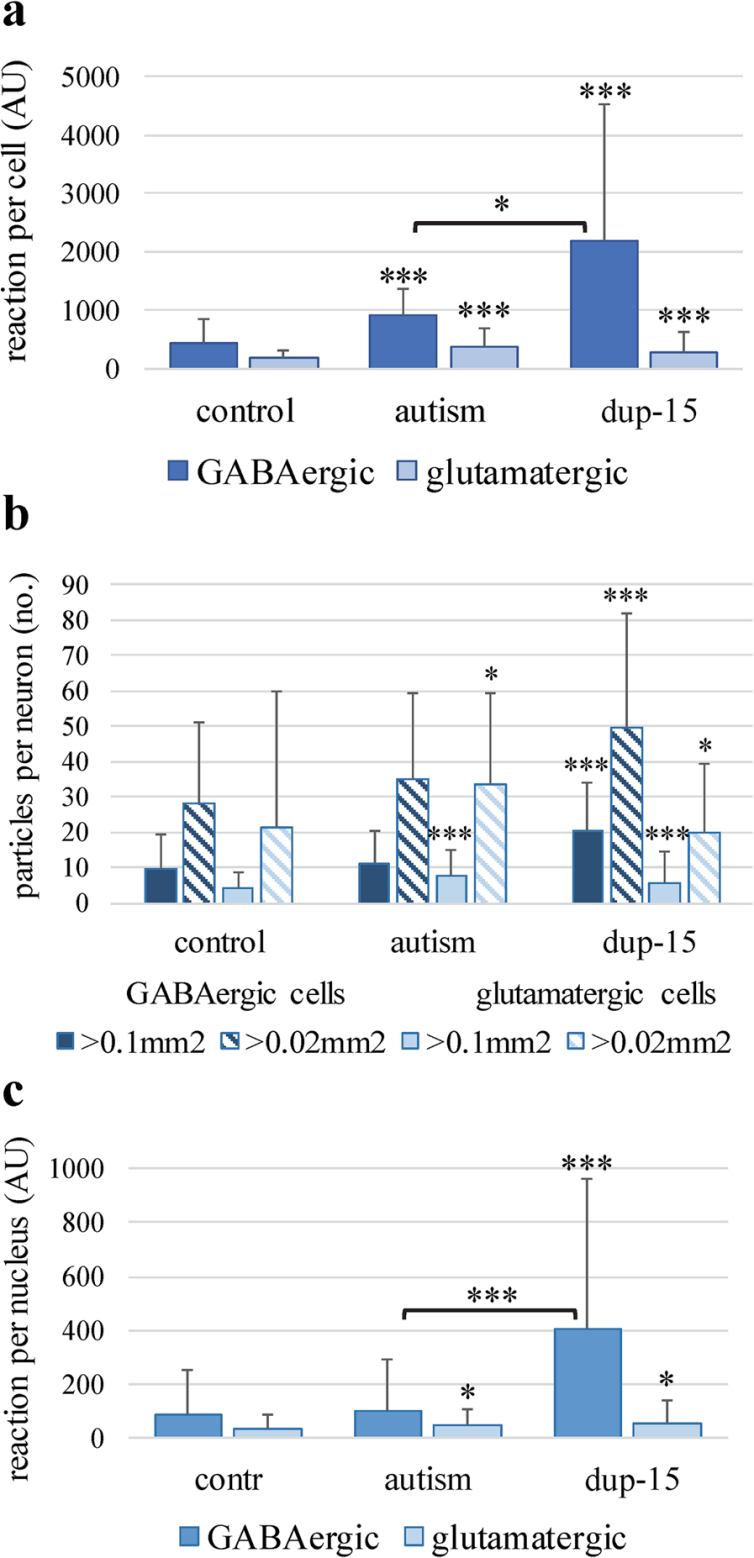
Immunoreaction for N-tr-Aβ measured in GABAergic and glutamatergic neurons in control, idiopathic autism (autism) and dup-15 groups. The graphs demonstrate the intensity of immunoreaction in arbitrary units (AU) in the perikaryon (**a**), numerical density of neuronal deposits (**b**), and nuclear load of N-tr-Aβ (**c**). The bars represent average ± SD; comparisons versus the appropriate control: * *p* < 0.05, *** *p* < 0.001

The prefrontal cortex neurons belong to one of two major populations: glutamatergic and GABAergic. The average cross section areas of the GABAergic neurons were significantly smaller than glutamatergic neurons (82 and 85% of the latter population in the control and autistic groups, respectively). The intracellular mAb 4G8 reaction had the morphology of condensed deposits of diameters between 0.08 μm to 2.5 μm. The immunoreactive cells were more frequent and the reaction was more intense in the autism (not shown) and dup-15 groups than in controls (Fig. 3). The frequency of neurons with an intensity of Aβ immunoreaction that exceeded 2 standard deviations over the average calculated for the control group was 5.1% in the control group, and 9.3 and 25.5% in the idiopathic autism and dup-15 with autism, respectively. The numbers of the N-tr-Aβ granules in neurons in individual subjects varied from no reaction to multiple granules in the neuronal perikarya in every group tested. The average intensities of the immunoreactions with mAb 4G8 per whole cell cross-section were significantly higher in the GABAergic than in glutamatergic cells in all studied groups (Fig. 4a). The cellular load of N-tr-Aβ in GABAergic neurons was significantly higher in autism than in control (*p* < 0.001) and was significantly higher in dup-15 than in the control (*p* < 0.001) and idiopathic autism groups (*p* < 0.05). In glutamatergic neurons the N-tr-Aβ load was significantly higher in autism and dup-15 than in controls (*p* < 0.001) (Fig. 4a). The numbers of cytoplasmic immunoreactive profiles in GABAergic neurons were significantly higher in dup-15 than in controls (*p* < 0.001), while among glutamatergic neurons cytoplasmic immunoreactive profiles of a size exceeding 0.1μm^2^ and 0.02 μm^2^ were both significantly more numerous in autism and dup-15 than in controls (*p* < 0.001 and *p* < 0.05, respectively), (Fig. 4b). Thioflavin S staining did not show fibrillar material in neurons (not shown).

The neuronal expression of GAD67 appeared to be less intense in cells containing more abundant N-tr-Aβ deposits, as visible in the confocal images with more than one GABAergic neuron present (Fig. 3, d15-magn). The intracellular load of N-tr-Aβ and GAD67/65 immunostaining intensity for the GABAergic neurons from the microscopic fields that contained two or three GABAergic cells which thus could be directly compared, were inversely correlated (Pearson’s correlation coefficient r = − 0.398).

Deposits of N-tr-Aβ were detected not only in the cytoplasm but also in the nuclei of GABAergic (Fig. 3, d15-magn) and glutamatergic neurons (not shown). The total nuclear immunoreaction intensities were significantly higher in GABAergic than glutamatergic cells in controls (*p* < 0.001), autism (*p* < 0.05), and dup-15 (*p* < 0.001), and among the GABAergic neurons were significantly higher in dup-15 than in controls and in autism (*p* < 0.001, and *p* < 0.01, respectively, Fig. 4c). The nuclei of glutamatergic neurons contained significantly more N-tr-Aβ in the autism and dup-15 groups than in controls (*p* < 0.05 for both comparisons), while there was no difference between the autism and dup-15 groups (Fig. 4c).

### N-tr-Aβ in subpopulations of GABAergic neurons

**Fig. 5:**
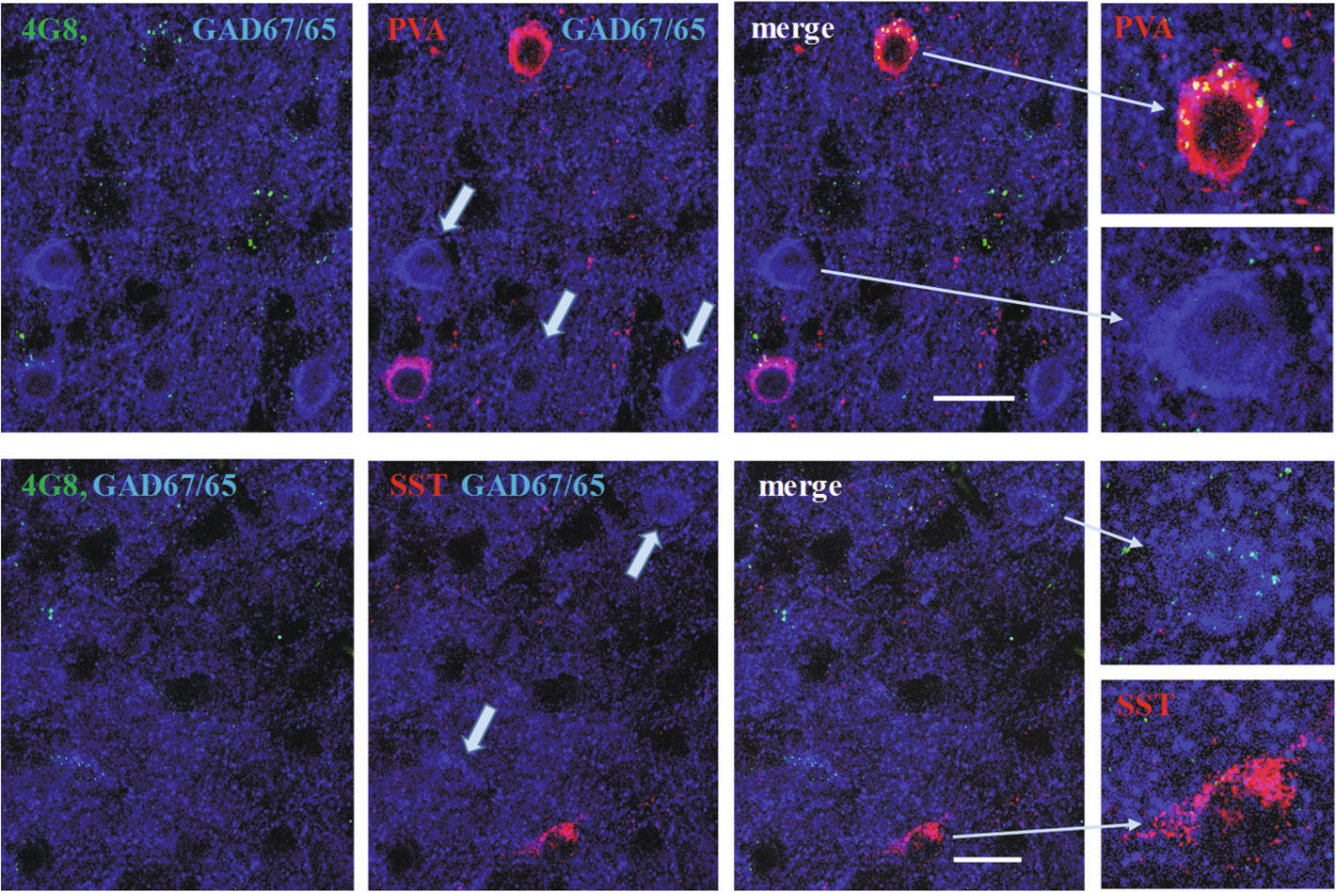
Intraneuronal N-tr-Aβ detected with the mAb 4G8, in subpopulations of GABAergic neurons expressing parvalbumin (PVA) and somatostatin (SST) shown in triple immunostainings in a 10-year-old dup-15/autism individual. GABAergic neurons (arrows) and synapses were stained blue for GAD67 and GAD65, respectively. All the cells expressing parvalbumin were GAD65/67 positive and appeared to be cells with a high load of N-tr-Aβ, while the SST-expressing neurons had low levels of N-tr-Aβ deposits. The scale bars show 20 μm

**Fig. 6:**
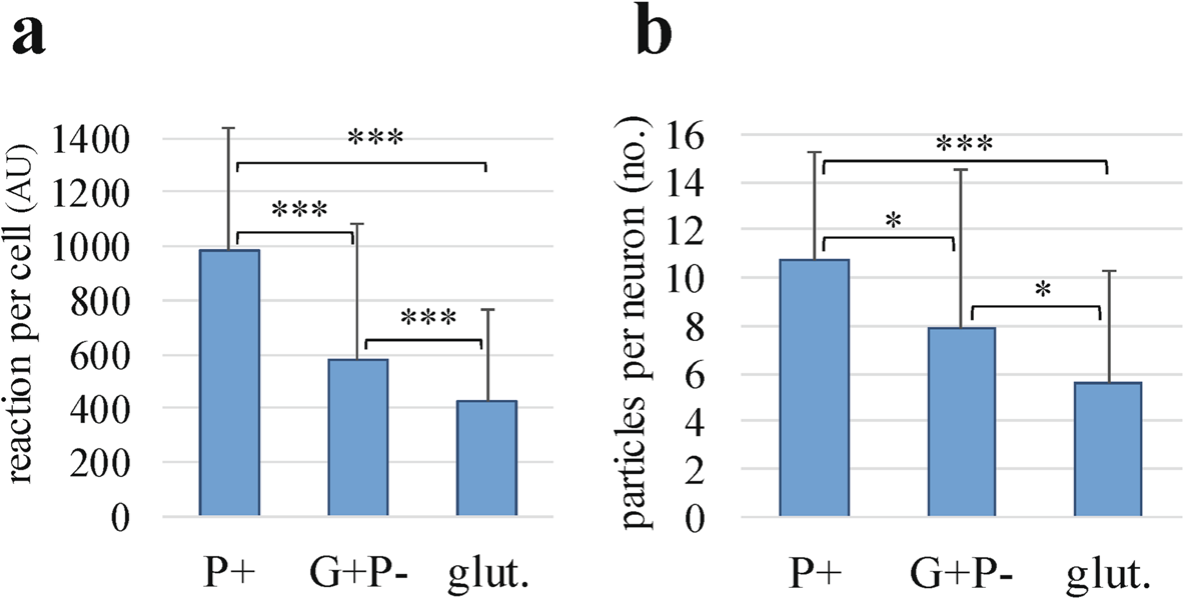
Quantification of N-tr-Aβ load measured as immunofluorescence intensity per neuron (**a**) and cellular Aβ-immunoreactive particles larger than 0.1 μm^2^ (**b**) in subpopulations of GABAergic neurons expressing parvalbumin (P^+^), GABAergic neurons PVA^**−**^ (G^**+**^P^−^), and glutamatergic neurons (glut.). The bars represent average AU values ± SD (* *p* < 0.05, *** *p* < 0.001)

The increased intensity of the immunoreaction for N-tr-Aβ in autism and in dup-15 varied greatly among individual GABAergic neurons, suggesting the existence of differentially affected neuronal subgroups. In this study the most frequent GABAergic neurons expressing PVA or SST were identified in two series of triple immunostainings, which combined labeling for GAD67/65, with mAb 4G8 and with immunostaining for either PVA or SST, without staining nuclei. The use of secondary antibodies labelled with Alexa647 or with NL637 gave a similar pattern of immunostaining for GAD65/67 and a similar quality of images. All the cells expressing parvalbumin were GAD65/67 positive neurons and appeared to contain high loads of N-tr-Aβ while GABAergic neurons the were PVA-negative (most of which are SST^+^) and neurons expressing SST revealed low levels of N-tr-Aβ load as revealed by the intensity of immunostaining and numbers of Aβ-immunoreactive particles (Figs. 5 and 6). The N-tr-Aβ load in cells of both subpopulations varied significantly, as demonstrated by relatively large SD values.

### N-tr-Aβ accumulation in GABAergic synapses and in neuropil

**Fig. 7:**
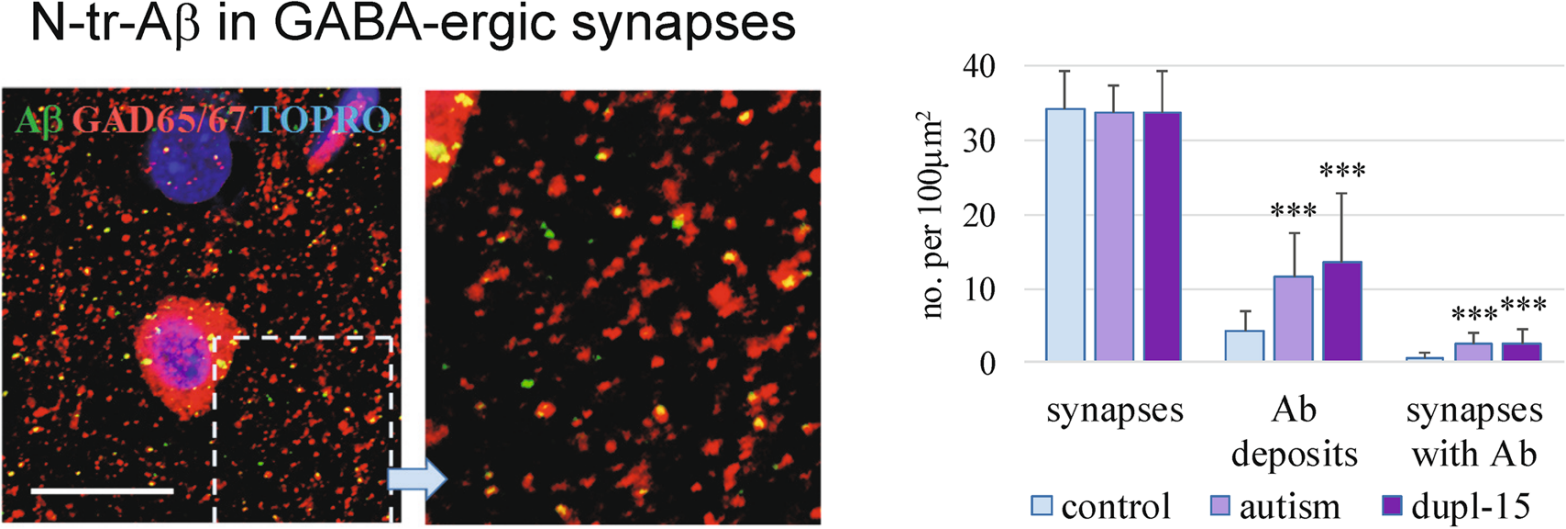
GABAergic synapses and N-tr-Aβ deposits in the neuropil in dup-15 with autism. The image shows an area rich in synapses with N-tr-Aβ deposits. The scale bar shows 20 μm. The numerical densities of Aβ-immunoreactive profiles of a diameter equal to at least 0.1 μm^2^ calculated per 100μm^2^ and numerical densities of GABAergic synapses colocalized with Aβ-immunoreactive profiles were significantly higher in autism and dup-15 groups than in control (*** *p* < 0.001)

The numerical density of GABAergic synapses in neuropil in the control group was 34.06 ± 4.19 per 100μm^2^ and was almost identical in the autism and dup-15 groups. The Aβ-immunoreactive profiles in the neuropil were significantly more frequent in autism and dup-15 than in controls. An area particularly abundant in Aβ-reactivity in the neuropil in dup-15 autism is shown in Fig. 7 (left panel). The numerical densities of the Aβ-immunoreactive profiles of the area equal to at least 0.1 μm^2^ per 100μm^2^ of tissue area were significantly higher in autism and dup-15 subjects than in controls (*p* < 0.001 in both comparisons), and there was no difference between autism and dup-15. Numerical density of GABAergic synapses colocalized with Aβ-immunoreactive profiles in control group was 0.51 per 100μm^2^ while the values were significantly higher in autism and dup-15 groups, equal to 2.51 and 2.63, respectively (*p* < 0.001 in both comparisons versus control) (Fig. 7, graph). These results show that 7.5 and 7.9% of GABAergic synapses in autism and in dup-15, respectively, contained N-tr-Aβ deposits, versus 1.5% in controls.

### Aβ-pE11 in GABAergic and glutamatergic neurons

**Fig. 8:**
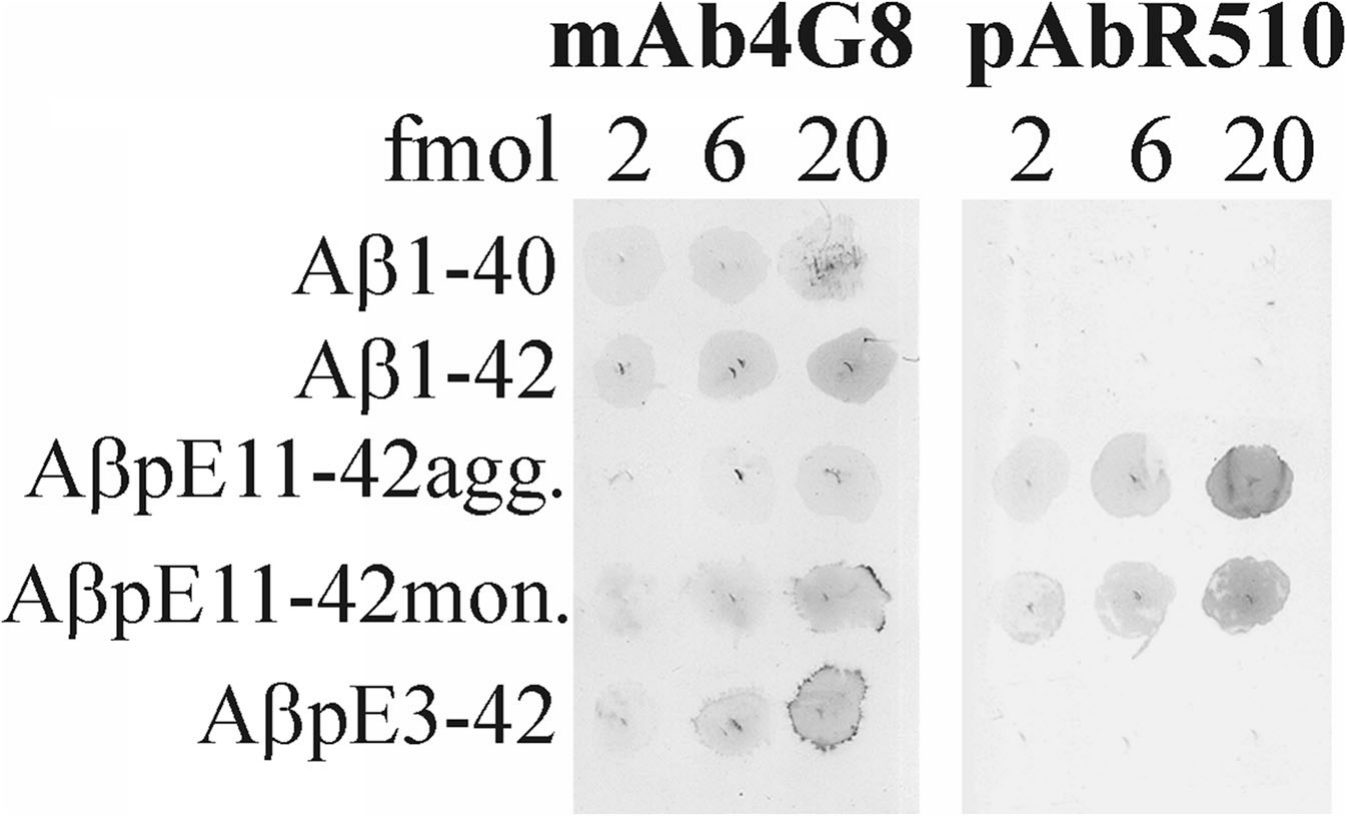
The reactivity of the rabbit polyclonal affinity purified antibody R510 (pAb R510) with synthetic Aβ-pE11 and other Aβ peptides tested by dot blotting. The intensities of the antibody reactions with Aβ-pE11 peptide were proportional to the amounts of the peptide in the 2–20 fmol range. There was no reaction with Aβ1–40, Aβ1–42 and AβpE3–42. The pAbR510 reacted similarly with oligomerized Aβ-pE11 peptide (AβpE11–42agg.) and the peptide freshly diluted from HFIP stock solution (AβpE3–42mon.). Oligomerization of the peptide reduced its reaction with mAb 4G8, used as the refence antibody

The rabbit polyclonal affinity purified antibody R510 showed optimal reaction with synthetic Aβ-pE11 in the concentrations of 0.05 and 0.2 μg/ml, as tested by ELISA and dot blotting. The reaction of the antibody with the peptide in ELISA was linear in the range between 5 and 1000 pg/ml of the peptide. The threshold of the peptide detection by dot blotting was about 1 fmol of Aβ-pE11. The intensity of the antibody reactions with Aβ-pE11 peptide was proportional to the amounts of the peptide in the range from 2 to 20 fmols, and the antibody did not react with Aβ1–40, Aβ1–42 and AβpE3–42 in ELISA and dot blotting (Fig. 8). Denaturation of the peptide by boiling the membrane had no effect on detection of Aβ-pE11 with R510. The reaction of pAb R510 was not affected by oligomerization of the peptide, as indicated by similar reactivity with the Aβ-pE11 peptide freshly diluted from HFIP stock solution and the peptide allowed to oligomerize. Oligomerization of the peptide reduced its reaction with mAb 4G8 (Fig. 8).

**Fig. 9:**
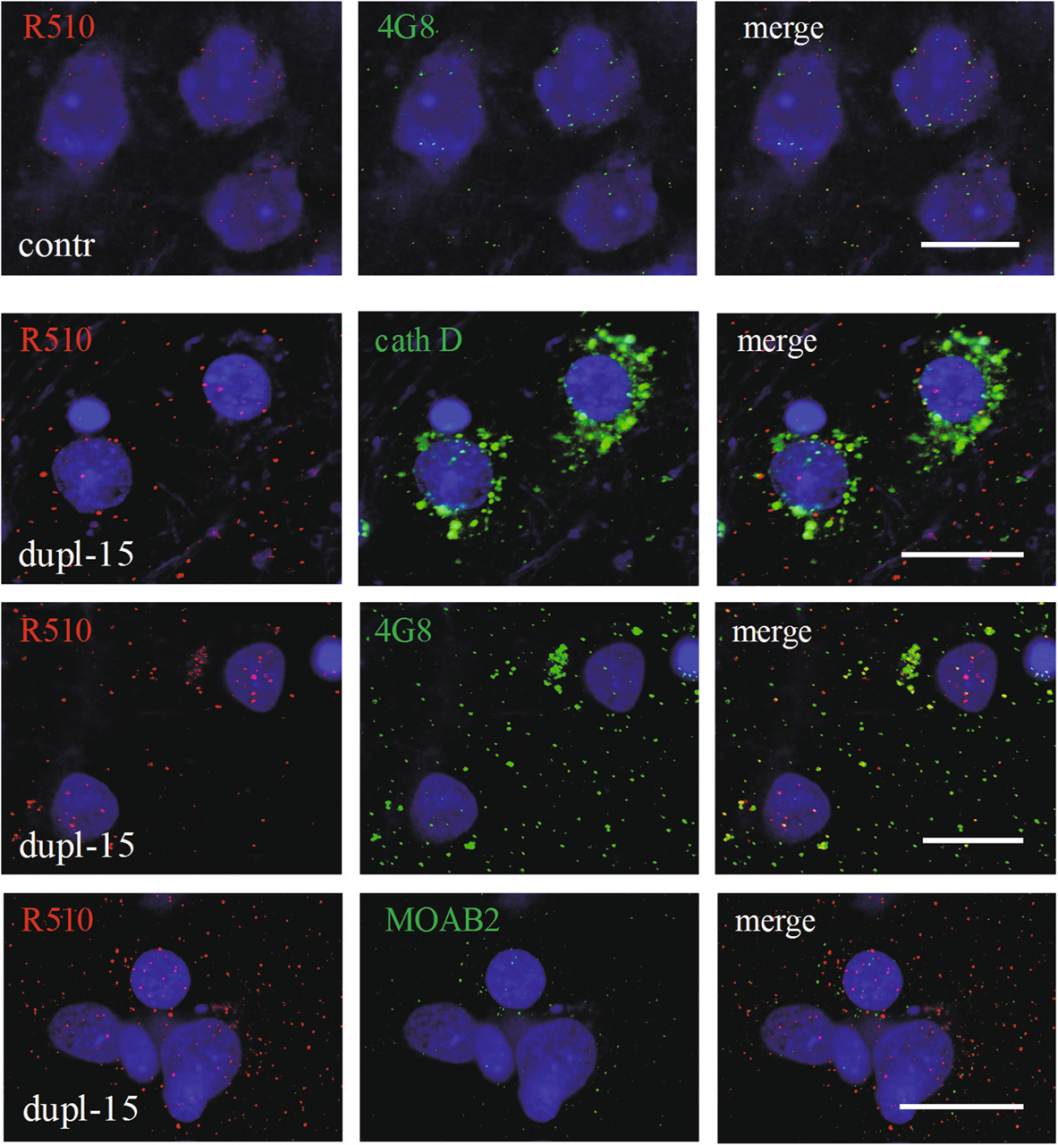
Aβ-pE11 (R510) in a control, 8 years old, and dup-15 (10-year-old and 15-year-old). The immunoreaction is almost negligible in the control, while in dupl-15 numerous small profiles are present in the cytoplasm and nucleus. The reaction is in part localized in lysosomes, detected by the reaction for Cathepsin D (cath D), and is partially co-localized with the immunostainings with mAb 4G8 and mAb MOAB2. A significant fraction of the reactivity for Aβ-pE11 is visible in the nucleus. The scale bars show 20 μm

**Fig. 10:**
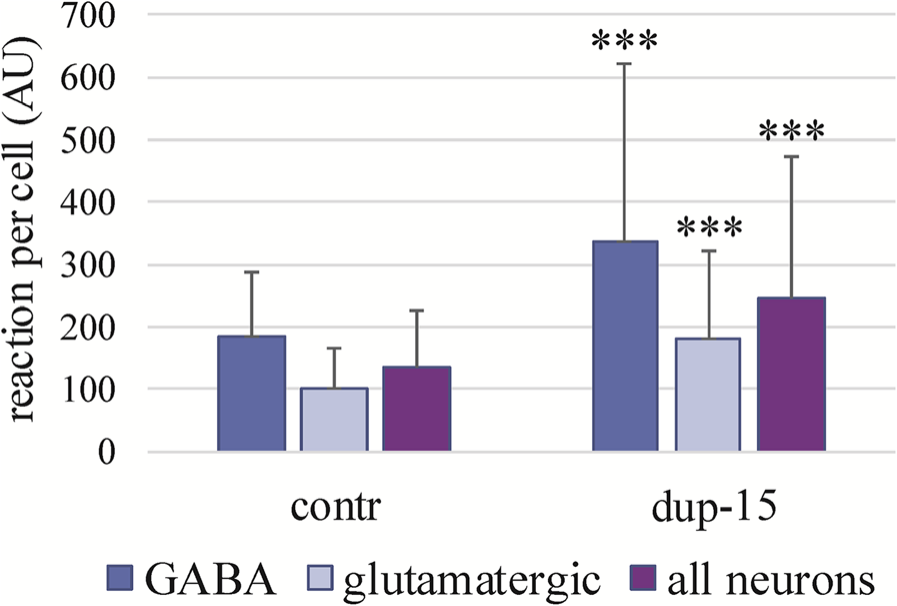
Intensities of the Aβ-pE11 immunoreaction in the GABAergic, glutamatergic and all measured neurons in control and dup-15 autism. The bars represent average AU values ± SD; statistical significance versus same type of cells in the control group: *** *p* < 0.001

Immunoreactivity for Aβ-pE11 in the brain sections was scanty in control brains but in dup-15 with autism was visible as granules in the cells, neuropil and in the walls of arteries and arterioles. The reaction was blocked by preabsorption of the antibody with the AβpE11–40 peptide. The Aβ-pE11 granules were detected in the neurons’ cytoplasm and nuclei; only a minor part of the Aβ-pE11 immunoreactivity was located inside Cathepsin D-positive lysosomes (Fig. 9). Most of the Aβ-pE11 immunoreactivity was co-localized with mAb 4G8 reactivity but was only partially co-localized with the reactivity for Aβ detected with mAb MOAB2 (Fig. 9). The Aβ-pE11 immunoreactivity was more intense in GABAergic than in glutamatergic neurons and the reaction in both neuronal populations was significantly higher in dup-15 than in controls (Fig. 10).

## Discussion

Our previous studies showed neuronal accumulation of N-tr-Aβ in idiopathic autism and in dup-15 with autism [48] and demonstrated almost complete co-localization of neuronal N-tr-Aβ deposits with markers of oxidative stress [16]. The morphological analysis of N-tr-Aβ deposits and oxidatively modified lipids suggested that N-tr-Aβ accumulation initiates oxidative stress. Hence, according to our hypothesis, intraneuronal deposition of N-tr-Aβ in autism in childhood causes enhanced formation of oxygen free radicals and lipid peroxidation products, which leads to the further formation of Aβ in a self-enhancing vicious circle contributing to neuron dysfunction in autism [16]. Now we demonstrate that in idiopathic and dup-15 autism this pathological process targets a specific subpopulation of GABAergic neurons, those expressing PVA. We also suggest that accumulation of N-tr-Aβ in GABAergic cells and synapses is a significant contributor to dysfunction of the GABAergic system that has been reported in autism [7, 13].

Detection of Aβ by the immunohistochemical method was based here on the use of the monoclonal antibodies 4G8 and 6E10, which may also bind full-length APP and its fragments, as reported previously [51]. However, in formalin-fixed and PEG-embedded human brains, the reaction is limited to Aβ [14–16, 48], as demonstrated also here in the double immunostainings (Fig. 1), which combine mAb 4G8 or 6E10 with APP-specific antibody R57.

The antibodies specific for the Aβ40 and Aβ42 species used for Aβ detection by immunoblotting do not react with APP or APP fragments after cleavage by α- or β-secretases, as they recognize the epitopes only when exposed as C-terminus. The usefulness of these antibodies in immunohistochemical staining in fixed sections, however, is limited, because of a lower sensitivity than mAb 4G8 — probably due to organization of Aβ species in the brain into complexes, oligomers, and aggregates (Fig. 2). Aβ peptide oligomerization reduces accessibility of the epitopes for the C-terminal–specific antibodies while preserving the reactivity with mAb 4G8 [19]. Polymorphism of Aβ aggregation states, dependent on peptide species and oligomerization/aggregation conditions, is known to affect the reactivity with numerous antibodies, including mAbs 4G8 and 6E10 [19]. It remains to be established if any particular Aβ oligomerization pattern is associated with specific neuron and synapse subpopulations in autism. The presence of lipofuscin, which is abundant in some neurons in autism [31], may generate problems with non-specific antibody binding. Thus, for detection of specific reactions essential are: optimization of the staining protocol and digital image collecting, and verification of fluorescence co-localizations in all three channels. The findings that mAb 4G8 and the Aβ-42– specific antibody R226 label only a fraction of autofluorescent granules, none of which are immunostained with mAb 6E10 (Suppl Fig. 1), indicate that our immunostaining protocol successfully prevents non-specific antibody binding in brain sections. The method of measurements of the N-tr-Aβ load that we applied here — immunohistochemistry, followed by confocal microscopy digital imaging and Image J analysis — has been shown to allow a reliable protein quantification in the model of cytochrome C aliquots embedded in gelatin [5].

### Accumulation of N-tr-Aβ in GABAergic neurons

The loads of N-tr-Aβ in GABAergic neurons and in GABAergic synapses in prefrontal cortex in idiopathic and dup-15 autism significantly exceed those found in controls. The mechanisms responsible for the observed accumulation of N-tr-Aβ may include altered processing of APP [2], as well as a decreased peptide clearance that involves transport through the perivascular drainage system and local enzymatic degradation, particularly by IDE, endothelin-converting enzymes (ECE)-1 and ECE-2, and neprilysin [37]. The two latter enzymes are mainly expressed in GABAergic neurons: ECE-2 primarily in SST-expressing neurons and synaptosomes, and neprilysin — mostly in synapses of the PVA-expressing interneurons. Hence, synapses of GABAergic neurons were suggested to be the sites of Aβ degradation [37]. Accumulation of N-tr-Aβ mainly in the PVA^**+**^ but not the SST^**+**^ subpopulation suggests that dysregulation of neprilysin expression in the former subpopulation might be a part of the pathomechanism of the observed accumulation of N-tr-Aβ in autism. Neprilysin is an important protective factor for neurons and neuronal progenitor cells against the damaging effects of Aβ [36].

Several pathophysiological consequences can emerge from the accumulation of Aβ in neurons, which have mainly been studied in the Alzheimer’s disease context. It should be noted that there is little knowledge about the effects of N-tr-Aβ and particularly Aβ-pE11, and that peptides’ truncation and N-terminal modification may significantly alter their biological effects. Soluble Aβ oligomers, even in low nanomolar concentrations, increase neuronal excitability by disrupting glutamatergic/GABAergic balance, thereby impairing synaptic plasticity [30]. Aβ injected into the hippocampus depresses the functional activity of GABAergic neurons responsible for the propagation of the theta rhythm without causing any actual cell damage [45]. Intraneuronal accumulation of Aβ peptides leads to a deep learning deficit detected in animal models, the mechanism of which is associated with a reduced nuclear translocation of the CREB co-activator, CRTC1, and decreased expression of the CRTC1-dependent genes associated with synaptic plasticity: Arc, c-fos, Egr1, and Bdnf [50].

Our finding of lower GAD67 in neurons that contain a high load of N-tr-Aβ suggests a reduced production of the GABA neuromediator and may signal a dysfunction of this fraction of GABAergic cells. Deficiency of GAD67 levels in PVA interneurons results in increased excitability of pyramidal cells and cortical dysfunction [29]. Reduction of the levels of GAD67 protein leading to a selective dysfunction of GABAergic interneurons can be induced by excessive stress during early development, as detected in a rat model of chronic unpredictable stress [3], and prenatal exposure to maternal stress specifically depresses precursors of PVA^**+**^ GABAergic interneurons [44]. These changes may be substantial in the pathophysiology of various stress-related disorders, including autism — known to be associated with prenatal stress and maternal immune dysregulation (reviewed: [6, 28]). We hypothesize that oxidative stress initiated by accumulation of N-tr-Aβ in neurons [16] may be responsible for most, if not all, of the above pathomechanisms.

### Nuclear N-tr-Aβ

In this study, the N-tr-Aβ–immunoreactive granules were also detected in the nucleus. In idiopathic and dup-15 autism, the nuclei of neurons contained between 14 and 20% of the total neuronal load of N-tr-Aβ. Full-length Aβ has been detected previously in the nucleus by biochemical methods, confocal microscopy, and electron microscopy in cultured neuroblastoma cells that internalized Aβ [4]. Aβ1–42 appears to have a role in nuclear signaling that is distinct from that of C-terminal APP, by specifically interacting as a repressor of gene transcription with LRP1 and KAI1 promoters. The nuclear translocation of Aβ1–42 impacts the regulation of genes, of which the most studied are the genes important in the context Alzheimer disease pathogenesis [4]. Aβ accumulation in neurons may repress the expression of multiple genes linked to synaptic plasticity, e.g., Arc, Nur77, and Zif268, in mouse models [11]. Zif268 in turn may regulate expression of GAD67, as the GAD67 promoter region contains a Zif268-binding site. Thus, accumulation of Aβ may affect genes’ expression also indirectly, e.g., through regulation of the gene Zif268, the equivalent of which in humans is the *EGR1* gene. Deficient EGR1 mRNA expression was detected in schizophrenia and was correlated with significantly lower levels of GAD67 [27]. Aβ1–42 in the nucleus of cortical neurons may also affect gene expression through a newly discovered mechanism: by affecting expression of miRNAs, the regulatory short RNA molecules [12]. It should be stressed that little is known about the effects on nuclear functions of N-tr-Aβ and pyroglutamate modified at glutamate-11; yet their nuclear presence in autism suggests they may also act as regulators of transcription in some neurons and possibly also in glia.

### Functional consequences of N-tr-Aβ in the PVA^**+**^ subpopulation of GABAergic neurons

The deposits of N-tr-Aβ and pyroglutamate-modified Aβ-pE11 were found primarily in the PVA^**+**^ subpopulation of GABAergic neurons. Inhibitory synapses of the PVA^**+**^ and SST^**+**^ GABAergic neurons are regulated by excitatory neurons through different postsynaptic proteins — either the L-type or R-type calcium channels, respectively [21], are regulated through distinct acetycholine receptor modulators [10] and have distinct effects on spatial working memory [24]. The fast-spiking parvalbumin interneurons in the medial prefrontal cortex appear to be involved in coordination of the activity in the local network during goal-driven attention processing [25]. Dysfunctions of the PVA^**+**^ GABAergic interneurons in the prefrontal cortex have been linked to cognitive deficits in schizophrenia [35] and other psychiatric disorders [10]. A significantly reduced density of the PVA^**+**^ neurons, but not the interneurons expressing calbindin or calretinin, was reported in the prefrontal cortex in autism as compared to control subjects [18].

We found a significantly higher accumulation of N-tr-Aβ in the PVA^**+**^ neurons; yet there was a substantial variability in the peptide load in individual cells in this subpopulation. The PVA^**+**^ neurons in the prefrontal and frontal cortex represent a diverse population that consists of basket and chandelier cells that in layer 3 form a circuitry with pyramidal cells. Thus, the variability we observed may represent either distinct functional subpopulations of PVA^**+**^ cells, or distinct stages of N-tr-Aβ accumulation.

Several differences in the accumulation of N-tr-Aβ and its pyroglutamate-modified form have been detected here between idiopathic autism and dup-15 with autism. These differences may result from the fact that human chromosome 15q11–13 contains a cluster of three GABAA receptor subunit (GABR) genes, GABRB3, GABRA5, and GABRG3. Deletion or duplication of 15q11–13 GABR genes occurs in multiple human neurodevelopmental disorders, including Prader-Willi syndrome, Angelman syndrome, and autism. In humans, all three GABR genes are biallelically expressed, i.e., are not imprinted in normal human cortex. However, in autism, expression of one or more GABR genes is frequently monoallelic or strongly skewed allelic, indicating that epigenetic dysregulation of these genes without cytogenetic modifications may be relatively common in autism [20].

### N-tr-Aβ in GABAergic synapses

The presence of N-tr-Aβ deposits in the GABAergic synapses — according to our study, in as many as 7% or more in autism, both idiopathic and dup-15 — may be a marker of dysfunction of the GABAergic system in autism. Aβ in soluble and aggregated forms has already been postulated as being responsible for synapse dysfunction. In cultured neurons, endogenous Aβ42 binds to a subset of synapses — more to glutamatergic than to GABAergic ones [49], and aggregated Aβ may damage axon terminals, even though the GABAergic neurons appear to be less vulnerable to Aβ toxic effects than cholinergic and glutamatergic ones [8]. It should be noted, however, that the toxic effects of low, even picomolar, doses Aβ oligomers on neurons can be greatly enhanced by inflammatory response to infections during critical stages of embryonic development and early postnatal life, when activated microglia cause synapse damage and cognitive impairment [17]. This modification of microglia function may be significant in the context of autism pathogenesis, in which prenatal and early postnatal infections have been postulated as triggering factors for development of autism [28, 42].

Processing of APP yields several products of distinct, yet only partially known functions. Alterations of APP processing in autism result in higher levels of not only N-tr-Aβ but also secreted APP-α [2, 38, 43]. The latter product in the brain may further affect the GABAergic regulations by suppressing presynaptic vesicle release through direct binding of sAPP extension domain to the GABA type B receptor subunit 1a [39]. This may be another APP-related mechanism of GABAergic dysregulation in autism.

## Conclusion

We provide morphological evidence that accumulation of N-tr-Aβ, which previously has been linked to a local oxidative stress in idiopathic and dup-15 autism [16], mainly affects the parvalbumin-expressing subpopulation of GABAergic neurons. GABAergic synapses are also the site of N-tr-Aβ accumulation. We hypothesize that the PVA^**+**^ GABAergic neurons with a high load of N-tr-Aβ become dysfunctional and are responsible for dysregulation of the brain excitatory–inhibitory homeostasis in autism and lead to behavioral disorders. This process may be the target of new therapies.

## Supplementary information

**Additional file 1:** Figure S1. Prefrontal cortex in dupl-15 with autism, 10 years old, immunostained with mAb 4G8 reveals granular intraneuronal reactivity highly variable among individual cells with respect to number, size and intensity. Only a fraction of the 4G8 reaction was co-localized with autofluorescence (enhanced in the picture in the red channel) while some autofluorescent granules did not immunostain with mAb 4G8. There was almost no reaction with mAb 6E10.

## Data Availability

The datasets generated and analyzed in this study are available from the corresponding author on a reasonable request.

## Abbreviations

Aβ: Amyloid-β peptide
Aβ-pE11: Amyloid-β peptide with N-terminal pyroglutamate-11 modification
APP: A-β precursor protein
AU: Arbitrary unit
Dup-15: Chromosome 15 duplication (Dup15q11.2-q13) with autism
GAD: Glutamic acid decarboxylases
GABR: GABA receptor subunit
mAb: Monoclonal antibody
N-tr-Aβ: N-terminally truncated Aβ
PVA: Parvalbumin
SST: Somatostatin, neuropeptide
