# Supplementary figures and images for: Enhanced accumulation of N-terminally truncated Aβ with and without pyroglutamate-11 modification in parvalbumin-expressing GABAergic neurons in idiopathic and dup15q11.2-q13 autism
Source: Acta Neuropathol Commun. 2020 Apr 28;8:58. doi: 10.1186/s40478-020-00923-8 (PMC7189730; doi:10.1186/s40478-020-00923-8)

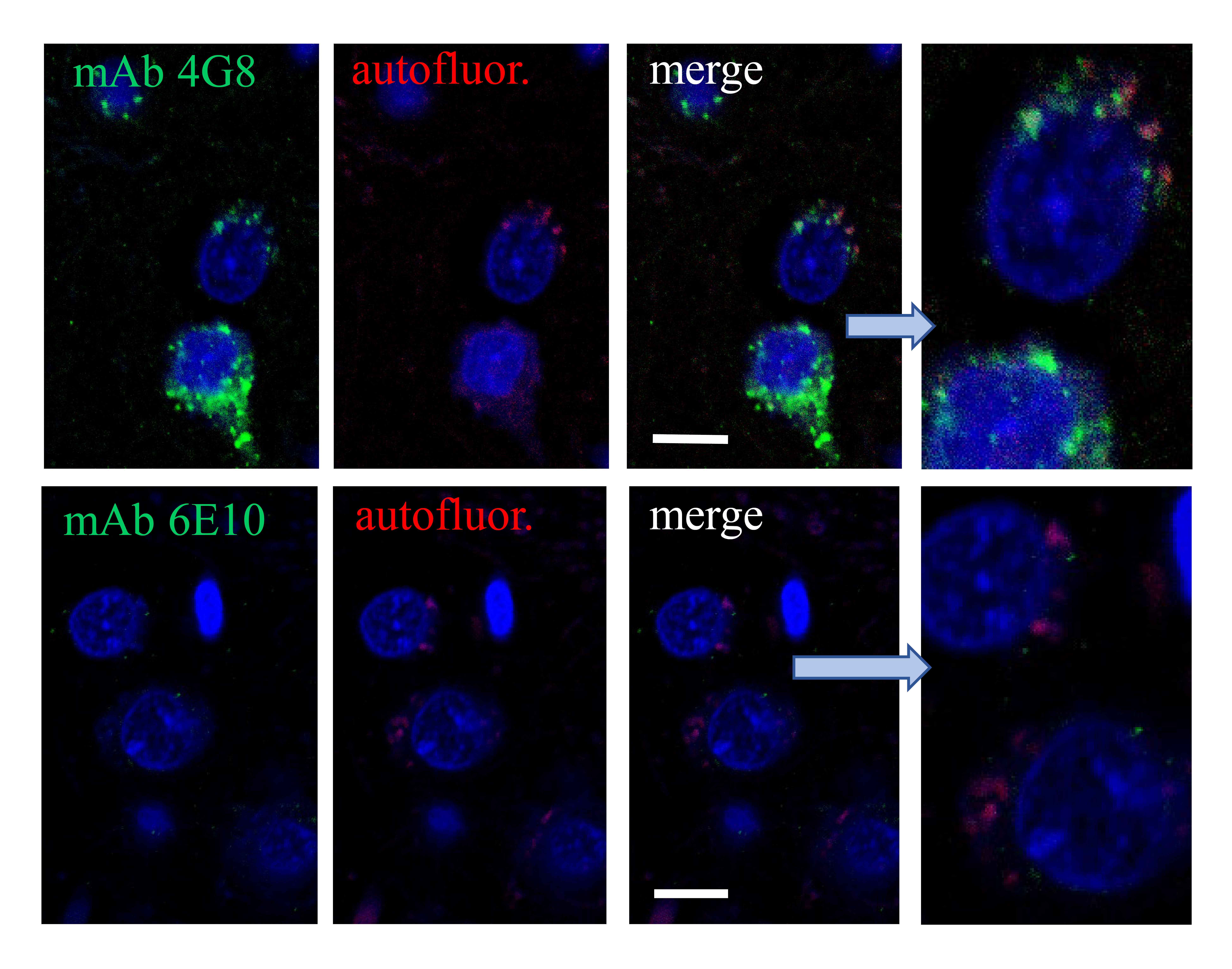

Supplement: Supplementary file 1 — Additional file 1: Figure S1. Prefrontal cortex in dupl-15 with autism, 10 years old, immunostained with mAb 4G8 reveals granular intraneuronal reactivity highly variable among individual cells with respect to number, size and intensity. Only a fraction of the 4G8 reaction was co-localized with autofluorescence (enhanced in the picture in the red channel) while some autofluorescent granules did not immunostain with mAb 4G8. There was almost no reaction with mAb 6E10. [file 40478_2020_923_MOESM1_ESM.tif]
